# Determinants of CD4 count and risk for death of HIV infected children under ART

**DOI:** 10.1038/s41598-022-10880-y

**Published:** 2022-04-27

**Authors:** Ayitenew Agegn Gwadu, Awoke Seyoum Tegegne

**Affiliations:** 1 Department of Statistics, Dire Dawa University, Dire Dawa, Ethiopia; 2 Department of Statistics, Bahir Dar University, Bahir Dar, Ethiopia

**Keywords:** Cancer, Cell biology, Drug discovery, Evolution, Neuroscience, Diseases, Health care, Medical research

## Abstract

The main objective of this study was to identify variables jointly affected for CD4 count and hazard time to death of HIV-infected children under ART at Felege Hiwot Referal and Specialized Hospital. A retrospective cohort study design was conducted on 202 HIV-infected children under ART whose follow-ups were from January 2014 up to December 2018. The descriptive statistics revealed that about 25.2% of HIV-infected children under ART in the study period(Jaunary 2014–December 2018) died and 74.8% were censored. The estimated association parameter in the joint model was − 0.8339 and statistically significant (*p* value = 0.025). There was a negative relationship between the two response variables namely CD4 count and the risk for death on HIV-positive children under treatment. The level of education of children's parents, level of disclosure of the disease, baseline CD4 count, functional status, and adherence level were statistically and significantly affected for the two response variables, CD4 count and risks for the death of children. Separate and joint models have been compared interims of standard error and the joint model had a small standard error as compared to the separate models. The small standard errors in joint models indicate that the joint model was better in detecting variables that affected the two responses in this regard. Health-related education should be conducted to parents of children for easy recovery of CD4 count and for reducing risks for the death of children.

## Introduction

About 36.7 million people are living with HIV globally and among these, about 2.1 million are children less than 15 years^1^. East and Southern Africa is the hardest region hit by HIV which means the region is the home of 19.4 million people living with the virus and this accounts for 6.2% of the world's population^1^.

Currently in Ethiopia, an estimated 1,216,908 people are living with HIV/AIDS, of which 79,871 are children^2^. Among people living with the virus, 397,818 (26,053 children) are under ART^2^.

Amhara region, one of the eleven regions in the country, comprises of 102,088 individuals under ART and this accounts for the highest proportion of ART users in the country. Hence, the region needs special attention to HIV-related problems such as the recovery of CD4 l count to highly active antiretroviral therapy(ART)^3^.

Previous research about HIV-infected patients focused on a separate analysis of the survival time of patients under ART^4^. A study is also conducted on the longitudinal repeated measures of CD4 cell count, considering the responses as a continuous variable and modeled using a linear mixed-effects model^5^. However, the distributions of count variables such as CD4 count are not continuous and not normally distributed, rather it is a count response variable and count regression models should be considered^6^. Linear mixed effect models for count response data give us inefficient estimates and the most appropriate longitudinal count models for efficient and unbiased estimates should be considered. Therefore, the current investigation was aimed to identify factors affecting jointly the CD4 count and risk for death on HIV-positive children under ART at Felege-Hiwot Comprehensive and Specialized Hospital, Amhara region, Ethiopia.

## Methods

### Study design

A retrospective cohort study design was conducted at Felege Hiwot Referal and Specialized Hospital, Bahir Dar, North West Ethiopia. The study was conducted in North-west Ethiopia (Amhara region).

### Source of data

In this study, secondary data collected by health staff for treatment purposes were used for data analysis. The data was collected from the medical chart of each HIV/AIDS patient in the ART clinic in the hospital.

### Study population

The study population consists of all HIV-positive children under 15 and started their ART treatment in January 2014–December 2018.

### Inclusion criteria

HIV/AIDS infected children under 15 who had at least two follow-ups in ART clinic for refilling their prescription and who have initiated their treatment from 1st January 2014 to 31st December 2018 at Felege Hiwot Referal and Specialized Hospital were eligible for the current study.

### Sample size determination

For the current investigation, there were 1304 HIV-positive children whose follow-ups were from January 2014 to December 2018. Among these, a random sample of 202 participants was selected using a stratified random sampling technique, considering their residence as strata.

### Variables under current investigation

The response variables under the current investigation were CD4 cell count and risk for death of HIV-positive children under treatment. The CD4 count was measured every six months. Hence, there were 10 visits for those patients with full records.

The predictor (independent) covariates under current investigation were sex (male, female), weight in kg, residence area (rural, urban), age in years, marital status of parents (living with a partner, living without a partner), education level of parents (non-educated, educated), level of disclosure of the disease by parents(yes, no), Baseline CD4 count, clinical stage (stage I, stage II, stage III, stage IV), functional status (ambulatory, bedridden, working), level of adherence (adherent, non-adherent), TB co-infection status (no, yes).

### Method of data analysis

In the current study, two models namely separate and joint models were considered for data analysis. Generalized linear mixed effect model for CD4 count and Cox proportional Hazard model for risk for death were conducted for data analysis^7^. The data were analyzed using SAS statistical software.

#### Consent for participants

Since the data was secondary and there was no chance of getting participants, consent for participants was not obtained from respondents. However, to get the secondary data from the hospital in the study area, and Ethical clearance certificate had been obtained from the Bahir Dar University ethics committee, Ethiopia with RefRCS/1412/2012. Hence, the Bahir Dar University Ethics Committee with RefRCS/1412/2012 waived the need for consent to participants.

#### Application of methods

All methods were performed in accordance with the relevant guidelines and regulations.

## Results and discussions

The baseline characteristics of respondents are summarized as indicated in Table 1. Table 1 indicates that about 13.9% of them were female children, 11.9% of the children’s parents were living without partners, 16.8% of the children’s parents were non-educated, 18.3% of them were urban children.

**Table 1:** Baseline characteristics of participants (n = 202).

Characteristics	Categories	Survival status		
		No. censored (%)	No. deaths (%)	Total (%)
Sex	Female	92 (45.5)	28 (13.9)	120 (59.4)
	Male	59 (29.2)	23 (11.4)	82 (40.6)
Marital status of parents	Without partner	60 (29.7)	24 (11.9)	84 (41.6)
	With partner	91 (45.0)	27 (13.4)	118 (58.4)
Educational level of parents	No education	48 (23.8)	34 (16.8)	82 (40.6)
	Education	103 (51.0)	17 (8.4)	120 (59.4)
Residence area	Urban	115 (56.9)	37 (18.3)	152 (75.2)
	Rural	36 (17.8)	14 (6.9)	50 (24.8)
Disclosure of the disease to other people, by parents	Yes	63 (31.2)	24 (11.9)	87 (43.1)
	No	88 (43.6)	27 (13.4)	115 (56.9)
WHO Clinical Stages	Stage I	18 (8.9)	6 (3.0)	24 (11.9)
	Stage II	48 (23.8)	9 (4.5)	57 (28.2)
	Stage III	63 (31.2)	21 (10.4)	84 (41.6)
	Stage IV	22 (10.9)	15 (7.4)	37 (18.3)
Functional status	Ambulatory	49 (24.3)	16 (7.9)	65 (32.2)
	Working	18 (8.9)	14 (6.9)	32 (15.8)
	Bed redden	84 (41.6)	21 (10.4)	105 (52.0)
TB-status	Positive	123 (60.9)	43 (21.3)	166 (82.2)
	Negative	28 (13.9)	8 (4.0)	36 (17.8)
Level of adherence	Adherent	42 (20.8	23 (11.4)	65 (32.2)
	Non-adherent	109 (54.0)	28 (13.9)	137 (67.8)
Drug type	d4t-3TC-NVP	34 (16.8)	11 (5.4)	45 (22.2)
	d4t-3TC-EFV	40 (19.8)	18 (8.9)	58 (28.7)
	AZT-3TC-NVP	38 (18.8)	12 (6.0)	50 (24.8)
	AZT-3TC-EFV	39 (19.3)	10 (5.0)	49 (24.3)

Continuous variable	n	Minimum	Maximum	Median (IQR)
Age in months	202	5	180	122.5 (114, 136)
Weight in kgs	202	13	37	22.5 (14, 31)

Similarly, 11.9% of the children disclosed the disease status, 10.4% of the HIV-positive children were bedridden, 21.3% of the participants in the current study were TB co-infected, 13% of the HIV-positive children started their ART were non-adherent to medication.

Finally, 51(25.2%) of the children died in the study period and the rest were censored. The median age of children was 12 months with interquartile range (114, 136) months and the median weight of children was 22.5 kg with interquartile range (14, 31) kg. Among the children who died in the study period, about 14% were females, 16.8% were non-educated family children, 13.4% were not disclosed the disease status by their parents, 10.4% were bedridden and 14% were non-adherent to ART medication. The parameter estimates for separate sub-model of CD4 count is indicated in Table 2.

**Table 2:** Results for the predictors of the longitudinal CD4 count sub-model (response = CD4 count).

Covariates	Estimate ($\beta )$	Std. Error	95% CI		*p* value
			Lower	Upper	
Intercepts	7.097	0.148	6.804	7.389	< 0.001*
Visiting time(number of visit of health institute by patients)	0.298	0.038	0.224	0.373	< 0.001*
Age	− 0.205	0.074	− 0.349	− 0.061	0.005*
Weight	0.259	0.073	0.115	0.403	0.004*
Baseline CD4 count	0.094	0.047	0.001	0.186	0.047*
**Education level (Ref = Non-educated)**					
Educated	0.160	0.074	0.016	0.305	0.030*
**Residence area (Ref = urban)**					
Rural	− 0.098	0.050	− 0.1967	0.002	0.051
**TB co-infection status (Ref. = no)**					
Yes	− 0.946	0.915	− 0.654	− 0.998	0.003*
**WHO Clinical stage (Ref = stage IV)**					
Stage 1	0.128	0.057	0.017	0.240	0.024*
Stage 2	0.216	0.091	0.039	0.393	0.017*
Stage 3	0.450	0.096	0.262	0.639	< .0.001*
**Functional status (Ref = Working)**					
Ambulatory	− 0.098	0.032	− 0.234	− 0.063	0.003*
Bedridden	− 0.287	0.105	− 0.494	− 0.181	0.006*
**Disclosure (ref = no )**					
Yes	0.286	0.083	0.124	0.447	0.005*
Dispersion Parameter	2.43				

Random effects	Estimates	95% CI			
		Lower	Upper		
Intercept ($Stdv\left({b}_{0i}\right))$	2.661	2.304	3.038		
Vis. time ($Stdv\left({b}_{1i}\right))$	0.188	0.163	0.225		
$\mathrm{cor}\left({\mathrm{b}}_{0\mathrm{i}},{\mathrm{b}}_{1\mathrm{i}}\right)$	− 0.092	− 0.080	− 0.106		

*Stands for statistically significant variable at 5% level of significant.

Table 2 indicates that visiting time(the number of times they visited the health institute), age of children, the weight of children, baseline CD4 count, level of education of parents, TB co-infection, WHO clinical stages, functional status, and disclosure status of the disease by the parents have significantly affected the response variable (CD4 count).

The positive sign of the estimated value of parameters($\beta$ value in Table 2) indicated that the increase of independent variable leads to the increase of CD4 count in HIV positive children while the negative sign of estimate indicates that the increase in predictor variable leads to a decrease in CD4 count. In the case of categorical variables like sex of patients, TB co-infected patients, the category with the positive sign of estimate of had greater CD4 cell count as compared to its counterpart and the category with the negative sign of estimate of had small CD4 count as compared to its counterpart.

As the number of visiting times of the patient increased by one unit, the expected CD4 count was also increased by 0.298, given that the other covariates constant ($\beta$ = 0.298, 95% CI (0.224, 0.373), *p* value < 0.001). Hence, as a patient closely follows up her/his medication treatment, the average /expected number of CD4 counts increased by 0.298 given the other covariates constant.

As age of achild increased by one month, the expected CD4 count was decreased by 0.205 ($\beta$ = − 0.205, 95% CI (− 0.349, − 0.061), *p* value = 0.005), given the other conditions constant.

Weight of a child had a significant contribution for the variation of CD4 count. Hence, as weight a child increased by one unit, the expected CD4 count was also increased by 0.259  ($\beta \hspace{0.17em}$= 0.259, 95% CI (0.115, 0.403), *p* value = 0.004), given the other conditions constant. Similarly, Baseline CD4 count positively affected current CD4 count, which means as baseline CD4 count increased by one cell/mm3, the expected current CD4 count was increased by 0.094 ($\beta \hspace{0.17em}$= 0.094, 95% CI (0.001, 0.186), *p* value = 0.047).

The expected(average) CD4 count of TB co-infected children was decreased by 0.946 as compared to those TB negative children, given the other conditions constant($\beta$ = − 0.946, 95% CI (− 0.654, − 0.998), *p* value = 0.003).

The expected CD4 count of HIV positive children with WHO status with Stage I was increased by 0.128 as compared to WHO Stage IV children ($\beta$ = 0.128, 95% CI (0.017, 0.240), *p* value = 0.024) given the other covariates constant.Similarly, the expected number of CD4 count for HIV positive children with WHO stage II was increased by 0.091 as compared to stage IV children ($\beta$ = 0.091, 95% CI (0.039, 0.393), *p* value = 0.017) given the other covariates constant.

The expected number of CD4 counts for ambulatory HIV-positive children was decreased by 0.098 as compared to HIV positive children with the working status given the other conditions constant ($\beta$ = − 0.098, 95% CI (− 0.234, − 0.063), *p* value = 0.003). Similarly, the expected number of CD4 counts for bedridden HIV-positive children was decreased by 0.287 as compared to HIV-positive children at working status ($\beta$ = − 0.098, 95% CI (− 0.234, − 0.063), *p* value = 0.003) given the other covariates constant.

The expected number of CD4 counts for HIV-positive children whose disease was disclosed by their parents was increased by 0.286 as compared to those HIV-positive children whose disease was not disclosed by parents ($\beta$ = 0.286, 95% CI (0.124, 0.447), *p* value = 0.005).

Predictors of CD4 count is summarized as follows; children with educated families, WHO clinical stage I, Stage II and Stage III, children with disclosed disease status, urban HIV-positive children, medication adherent children, and children with high baseline CD4 count were associated with higher expected CD4 count while, non-adherent to medication, children from rural areas, children from non-educated family, children who started their ART with small baseline CD4 count and those children whose disease not disclosed were associated to a low number of CD4 count.

Similar to Table 2, Table 3 also indicates the separate sub-model for parameter estimates of risk/hazard for the death of children. The education level of parents, clinical stages, level of adherence, functional status, disclosure of the disease, and TB co-infection status significantly affected the response variable (risk for death of HIV infected children) at 5% of the level of significance.

**Table 3:** Parameter estimates for risk for deaths (Cox proportional hazards sub-model for risk for death).

Covariates	Estimate	Std. Error	HR(95% CI)	*p* value
Weight	− 0.007	0.017	0.993 (0.961, 1.026)	0.671
Age	0.145	0.320	1.156 (0.141, 1.172)	0.650
**Education level (Ref = No education)**				
Education	− 0.946	0.379	0.388 (0.185, 0.817)	0.013*
Baseline CD4 count	− 0.021	0.321	0.979 (0.743, 0.999)	0.002*
**Clinical stage (Ref = stage IV)**				
Stage I	− 0.961	0.362	0.383 (0.188, 0.777)	0.008*
Stage II	− 1.372	0.4213	0.2536 (0.1110,0.5790)	0.0011*
Stage III	− 1.0146	0.460	0.3625 (0.1471, 0.8934)	0.0275*
**Adherence (Ref = non-adherent)**				
Adherent	− 1.3131	0.375	0.269 (0.1289, 0.5611)	0.005*
**Functional status (Ref = ambulatory)**				
Working	− 1.125	0.406	0.325 (0.146, 0.719)	0.006*
Bedridden	0.103	0.416	1.1085 (1.034, 1.812)	0.008*
**Disclosure (Ref = no)**				
Yes	− 1.394	0.433	0.248 (0.106, 0.579)	0.001*
**Sex (Ref = male)**				
Female	− 0.884	0.480	0.413 (0.161, 1.059)	0.066
**TB co-infection status (Ref. = no)**				
Yes	0.453	0.745	1.573 (1.353, 1.877)	0.005*

In interpreting the significant variables, the value of HR < 1 indicates that the predictor variable associated with this value and the variable of interest(risk for death) was negatively associated and the HR value > 1 indicates that the predictor variable corresponding to this value and risk for death were positively correlated.

Hence, The rate of hazard for the death of educated family children was decreased by 61.2% (1–0.388)*100 as compared with non-educated family children, keeping the other covariates constant (HR = 0.388, 95% CI (0.185, 0.817), *p* value = 0.013).

As baseline CD4 cell count increased by one cell/mm3, the rate of hazard/risk for death was decreased by 2.1% , keeping the other covariates constant (HR = 0.979, 95% CI 0.743, 0.999), *p* value = 0.002).

The rate of hazard/risk for death of adherent children was decreased by 73.1% as compared to non-adherent patients, given the other covariates constant (HR = 0.269, 95% CI (0.128, 0.579), *p* value = 0.005). Similarly, the rate of hazard/risk for death of TB-coinfected children was increased by 57.3% as compared to non TB co-infected children, given the other covariates constant (HR = 1.573, 95% CI (1.353, 1.877), *p* value = 0.005).

The rate of risk for death of HIV positive children whose WHO stage I was decreased by 61.2% as compared to those of HIV positive children whose WHO stage IV given the other covariates constant (HR = 0.383, 95% CI (0.188, 0.777), *p* value = 0.008). The rate of risk for death of HIV positive children with WHO stage II was decreased by 74.6% as compared to HIV positive children with WHO stage IV given the other covariates constant (HR = 0.254, 95% CI (0.111, 0.579), *p* value = 0.001).

The rate of risk for death of HIV-positive children with working functional status was decreased by 67.5% as compared to ambulatory children (HR = 0.325, 95% CI (0.146, 0.719), *p* value = 0.006). On the other hand, the rate of risk for death of bedridden children was increased by 10.9% as compared to bedridden children, given the other covariates constant (HR = 1.1085, 95% CI (1.2344, 1. 8120), *p* value = 0.008).

The rate of risk for death of children whose disease was disclosed by their parent was decreased by 76.2% as compared to those children whose disease was not disclosed, given the other covariates constant(HR = 0.248, 95% CI (0.106, 0.579), *p* value = 0.001).

Finally, the rate of risk for death of HIV/TB co-infected children was increased by 57.3% as compared to non-con-infected children, given the other covariates constant(HR = 1.573, 95% CI (1.353, 1. 877), *p* value = 0.005).

The potential joint predictors of CD4 count and risk for death in Table 4 revealed that baseline CD4 cell count, level of education, functional status of patients, and adherence level of prescribed medication significantly affected CD4 count and risk for death. The estimate of the association parameter ( ) in the survival sub-model under joint analysis was significantly different from zero and this leads to evidence of the association between the two outcomes. As baseline CD4 cell count of patients increased by one cell/mm3, the expected number of CD4 count was also increased by 0.0958 (*p* value = 0.0404) but the rate of risk for death of such patients was decreased by 0.4321(*p* value = 0.0021) keeping the other variables constant.

**Table 4:** Parameter estimates and Standard Errors under the joint modeling analysis for CD4 count and hazard to death.

Longitudinal process (CD4 count)				Survival process (hazard to death)			
Parameters	$\widehat{\beta }$	Se ($\widehat{\beta }$)	*p* value	Parameters	$\widehat{\beta }$	Se ($\widehat{\beta }$)	*p* value
Intercepts	6.806	0.137	< 0.001	Weight	− 0.001	0.017	0.935
Visiting time	0.289	0.038	< 0.01*	Age	0.093	0.319	0.061
Age	− 0.205	0.074	0.075	**Education level (ref = no education)**			
Weight	0.256	0.073	0.004*	Education	− 0.896	0.378	0.018*
BaselineCD4	0.096	0.047	0.040*	**Clinical stages (stage iv)**			
**Residence (Ref = Urban)**				Stage I	− 1.034	0.353	0.003 *
Rural	− 0.098	0.050	0.050	Stage II	− 1.388	0.419	0.001*
**Education level (Ref = No education)**				Stage III	− 1.035	0.460	0.024*
Educated	0.155	0.073	0.035*	**Adherence (Ref = non-adherent)**			
Stage I	0.126	0.057	0.027*	Adherent	− 1.159	0.364	0.001*
Stage II	0.239	0.089	0.007*	Baseline CD4	− 0.432	0.342	0.002*
Stage III	0.459	0.096	< 0.001*	**Functional status (Ref = ambulatory)**			
**Functional status (Ref = Ambulatory)**				Working	− 0.799	0.375	0.033*
Working	0.113	0.032	0.005*	Bedridden	1.024	0.402	0.011*
Bedridden	− 0.314	0.1034	0.003*				
**Adherence Level (Ref = Non-adherent)**				**Disclosure (Ref = no)**			
Adherent	0.247	0.082	0.031*	Yes	− 1.588	0.424	0.001*
**Disclosure (ref = no)**				**Sex (Ref = male)**			
Yes	0.287	0.082	0.005*	Female	− 1.099	0.466	0.068
**TB co-infection status (Ref. = No)**				**TB co-infection (Ref. = No)**			
Yes	− 0.464	0.007	0.003*	Yes	0.245	0.078	0.005*
Random effects	Std.dev			Association parameter (${\theta }_{0}$)	− 0.834	0.374	0.026
Intercept $\left({b}_{0i}\right)$	2.649						
Vis. time $\left({b}_{1i}\right)$	0.188						
$\mathrm{Cor}\left({\mathrm{b}}_{0\mathrm{i}},{\mathrm{b}}_{1\mathrm{i}}\right)$	− 0.0934						

*stands for statistically significant variable at 5% level of significance.

The expected number of CD4 count for adherent patients was increased by 0.247 cells per mm3 (*p* value = 0.0031) and the rate of hazard/risk for death for such patients was decreased by 68.6% ((1 − 0.318 )*100%)) (*p* value = 0.0005) as compared to non-adherent patients, given the other variables constant.

Functional status had also a significant effect on the variation of CD4 cell count and risk for death of HIV-positive children. Hence, the expected number of CD4 counts for patients at working status was increased by 0.113 (*p* value = 0.005) but the rate of risk for death was decreased by 0.799 (*p* value = 0.033) as compared to ambulatory patients, given the other variables constant.

TB co-infection statistically affected the two responses namely CD4 count and hazard to death. The expected number of CD4 count for HIV/TB co-infected patients was decreased by 0.464 (*p* value = 0.003) and the rate of hazard to death of such patients was increased by 27.8% (( ), *p* value = 0.0047)), as compared to HIV positive but TB negative status, given the other covariates constant.

Similarly, the expected number of CD4 count for patients who disclosed their disease to the community around them was increased by 0.287 (*p* value = 0.005) and the rate of hazard to death of patients who disclosed the disease was decreased by 1.588 (*p* value = 0.001) as compared to those patients who did not disclose their disease, keeping the other variables constant (refer to Table 4).

The estimates of the parameters of the separate and joint models are quite similar to each other but not identical. We compared separate and joint models based on standard errors computed in the two models in detecting the significant predictors for the variable of interest. The model with a smaller standard error is the better fit for the data. In the current investigation, we observed that the joint model had smaller standard errors for all significant predictors as compared to a separate model. The statistical significance of the association parameter is also evidence that the joint model was better than the separate models.

## Discussion

In our study, the association parameter statistically significant in the joint model indicates that the two responses were correlated, and shows that the joint model is a better fit for the data than the separate models. This finding was consistent with another study^8^ and the result showed that the statistical significance of the association parameter is evidence that the joint model is a better fit than the separate models. CD4 count and hazard to death were found to evolve differently between educated and non-educated parents of children. This might occur because as parents of patients become more educated, they may have better care of their children’s health and they may have enough understanding about ART this further leads the CD4 count may increase and leads to living for a long period of time^9^. Hence, the hazard to death for the educated patient is lower than non-educated patients^10^.

WHO clinical stages have significant effects on the variation of CD4 count and weight of patients. Hence, patients who started their treatment at an earlier stage can survive for a long period of time because of good recovery of the CD4 count^11^. This might make them able to take the treatment properly due to less replication of the virus in their body and due to this death from ART may be lower^12^.

The functional status of the HIV patients affected the long life and recovery of CD4 cell count. Hence, patients bedridden are at a high rate of hazard to death and recovery of CD4 cell count change. On the other hand, patients who are in working functional status can take prescribed medication by themselves on the time given by the health staff which leads to good recovery of CD4 count^13^.

Patients who disclosed their disease to the communities around them and to their parents can take pills at the prescribed time whether people are there or not. On the other hand, patients, who didn’t disclose their disease may not take pills when they are living with the other. Unable to take pills at a prescribed time leads to less recovery of CD4 count and further leads to death of patients within short periods of time^14^. Adherence to the prescribed medication has a significant association with the variation of CD4 count and hazard to death. This result is supported by previously conducted research^15^.

In current investigation, the predictors namely level of adherence, WHO stages, disclosure status, baseline CD4 count, functional status and TB co-infection palyed significant role for the variation of CD4 count and associated risk for death of HIV-positive children under ART.

As a recommendation, more emphasis should be given to bedridden and ambulatory HIV-positive children, for those patients who did not start their treatment on time (for patients whose WHO stage is stage 4). Special attention should be given to HIV/TB co-infected patients. In addition, health-related education should be given to non-adherent patients. Patients should be advised to disclose the disease to their parents as well as to the community around them.

This research is not without limitation, the research used secondary data with limited variables to be considered as predictors. Including additional variables as a predictor variable may provide additional information. This can be considered as a research gap.

## References

[1] Unaids J (2017). Fact Sheet—Latest Global and Regional Statistics on the Status of the AIDS Epidemic.

[2] Flores SA (2016). Shifting resources and focus to meet the goals of the National HIV/AIDS Strategy: The Enhanced Comprehensive HIV Prevention Planning project, 2010–2013. Public Health Rep..

[3] Tsegaye AT (2016). Predictors of treatment failure on second-line antiretroviral therapy among adults in northwest Ethiopia: a multicentre retrospective follow-up study. BMJ Open.

[4] Atnafu H, Wencheko E (2012). Factors affecting the survival of HIV-infected children after ART initiation in Bahir-Dar, Ethiopia. Ethiop. J. Health Dev..

[5] Adams M, Luguterah A (2013). Longitudinal analysis of change in CD4+ cell counts of HIV-1 patients on antiretroviral therapy (ART) in the Builsa district hospital. Eur. Sci. J..

[6] Allison PD (2005). Fixed Effects Regression Methods for Longitudinal Data Using SAS.

[7] West BT, Welch KB, Galecki AT (2014). Linear Mixed Models: A Practical Guide Using Statistical Software.

[8] Seid A (2014). Joint modeling of longitudinal CD4 cell counts and time-to-default from HAART treatment: a comparison of separate and joint models. Electron. J. Appl. Stat. Anal..

[9] Kebede MM, Zegeye DT, Zeleke BM (2015). Predictors of CD4 count changes after initiation of antiretroviral treatment in University of Gondar Hospital, Gondar in Ethiopia. Clin. Res. HIV/AIDS.

[10] Tadege M (2018). Time to death predictors of HIV/AIDS infected patients on antiretroviral therapy in Ethiopia. BMC. Res. Notes.

[11] Seyoum A, Zewotir T (2016). Quasi-Poisson versus negative binomial regression models in identifying factors affecting initial CD4 cell count change due to antiretroviral therapy administered to HIV-positive adults in north–West Ethiopia (Amhara region). AIDS Res. Ther..

[12] Lumbiganon P (2011). Survival of HIV-infected children: a cohort study from the Asia-Pacific region. J. Acquir. Immune Defic. Syndr..

[13] Gezie LD (2016). Predictors of CD4 count over time among HIV patients initiated ART in Felege Hiwot Referral Hospital, northwest Ethiopia: multilevel analysis. BMC. Res. Notes.

[14] Tekle G, Kassahun W, Gurmessa A (2016). Statistical analysis of CD4+ cell counts progression of HIV-posi tive patients enrolled in antiretroviral therapy at Hossana District Queen Elleni Mohamad Memorial Hospital, South Ethiopia. Biom. Biostat. Int. J..

[15] Guo X, Carlin BP (2004). Separate and joint modeling of longitudinal and event time data using standard computer packages. Am. Stat..

